# Music Therapy as a Tool to Unveil Musical Potential or Hidden Savant in Children with Autism: A Case Study

**DOI:** 10.3390/children11121543

**Published:** 2024-12-19

**Authors:** Mais Hatahet, Andrea Sárváry, Attila Sárváry

**Affiliations:** 1 Faculty of Health Sciences, University of Debrecen, 4400 Nyíregyháza, Hungary; hatahetmais@foh.unideb.hu; 2 Doctoral School of Health Sciences, University of Debrecen, 4032 Debrecen, Hungary; 3 Department of Psychology, Faculty of Health Sciences, University of Debrecen, 4400 Nyíregyháza, Hungary; sarvary.andrea@etk.unideb.hu; 4 Department of Integrative Health Sciences, Faculty of Health Sciences, University of Debrecen, 4400 Nyíregyháza, Hungary

**Keywords:** music therapy, savant syndrome, musical talent, autism spectrum disorder

## Abstract

**Background/Objectives**: Savant syndrome is a perplexing condition characterized by the exceptional abilities or talents of individuals with disabilities or low IQ. **Methods**: This study shows an individual case of a child with autism, detailing how music therapy may facilitate the discovery of musical abilities and how it can improve different areas of development, e.g., motor function, communication, social skills, and emotional expression. **Results**: The 17-year-old male (KH), diagnosed at the age of three with autism spectrum disorder (ASD) and having moderate abilities, exhibited no interest in music until the initiation of a music therapy program at the age of 11 years. KH consistently has repeated movements which impede his advancement in various tasks. This observation prompted the occupational therapist to purposefully incorporate this movement into piano training and recommend the initiation of music therapy sessions. He subsequently began utilizing the piano and demonstrated an extraordinary aptitude for musical note interpretation and creation. **Conclusions**: Occupational therapy may contribute to revealing hidden savant syndrome in children with ASD. Prolonged musical training has multiple impacts on motor functioning and multi-sensory perception, and it may also elicit favorable behavioral modifications in children with autism.

## 1. Introduction

Music is a creative and sensory art form that possesses unique therapeutic, mood-enhancing, and gratifying effects. Furthermore, it functions as the primary language for mother–child communication in articulating the human experience [1]. Anthropological studies of music provide several instances of its capacity to articulate emotions, concepts, and events [2].

The term “music savant” describes a person who possesses exceptional musical ability or genius, frequently in spite of difficulties or limits in other areas of life. Savant syndrome can present as exceptional musical skills that stand out in the setting of autism, such as the ability to play an instrument with little to no instruction, memorize difficult compositions, or possess an innate knowledge of musical theory [3]. Around 50% of savants are on the autistic spectrum, indicating a substantial overlap between the two conditions [4]. However, parents or family members may not immediately identify musical talent in their children; furthermore, if these talents are overlooked or not nurtured within a dynamic atmosphere, they may diminish over time [5].

The diagnostic criteria for savant syndrome were employed to characterize the exceptional abilities or talents of individuals with disabilities or low IQ. Individuals with pronounced exceptional abilities are more prone to exhibit autistic symptoms within the population possessing special abilities, but those with remarkable exceptional abilities in the autism spectrum disorder (ASD) population demonstrate less severe autistic behaviors [6]. Approximately 10% of children with ASD exhibit savant syndrome. Approximately fifty percent of those diagnosed with savant syndrome are autistic, while the remaining fifty percent possess various other forms of developmental disabilities [7].

Exceptional musical ability is a prominent expression of savant syndrome; however, other skills, including calendar calculation, drawing, arithmetic, mechanics, and memory, have also been documented. There is no comprehensive explanation for the elevated prevalence of savant syndrome in individuals with ASD [8].

Research indicates that individuals with dementia, ASD, developmental delays, or psychiatric disorders exhibit unique responses to music, and an increasing number of studies reveal that music significantly enhances social, physiological, and neurological responses in children with ASD [9]. Music therapy significantly activates multiple brain regions, and children with ASD may possess exceptional yet concealed musical abilities [10]. Furthermore, continuous music therapy combined with musical education for children with autism can significantly enhance musical abilities [11].

This case study presents a male patient’s experiences with music therapy and its impact on his emotional and social development, in addition to exploring his hidden music talent.

## 2. Case Report

### 2.1. Music Therapy Prompts Musical Skills

Our case: KH was born in 2007 after a normal delivery with no complications. At the age of three, he could articulate whole sentences but exhibited problems in reciprocal communication of his emotions, atypical social behaviors, and repetitive gestures. He was diagnosed with ASD by a child neurologist after having been examined several times by a rehabilitation therapist and clinical psychologist. After the autism diagnosis, his parents decided to go to Jordan in 2018 to receive intervention therapy at the Autism Academy of Jordan, as it is one of the best centers in the region.

To estimate the severity of the child’s autism, the Gilliam Autism Rating Scale—Third Edition (GARS-3) was used [12]. This scale can be used between ages 3 and 22 and provides four levels of probability of having ASD: level 0, with an Autism Index ≤ 54, “unlikely”; level 1, with an Autism Index between 55 and 70, requiring “minimal support”; level 2, with an Autism Index between 71 and 100, “very likely” and requiring substantial support; and level 3, with an Autism Index ≥ 101, “very likely” and requiring very substantial support. KH’s initial assessment on the GARS-3 indicated a score of 68 at level one, signifying a need for minimal assistance in August 2018.

One month later, the occupational therapist at the facility noticed that KH consistently exhibited repetitive movements, which impeded his ability to accomplish various tasks. Interestingly, one of his repetitive movements observed by the occupational therapist during states of irritation, excitement, happiness, or sadness involves him moving his fingers up and down as if playing an imaginary piano. This caught the therapist’s attention, prompting a recommendation to purposefully utilize this movement for piano training and to initiate music therapy sessions. Before the initiation of the music therapy program, he had little interest in music during his developmental phases, as mentioned by his parents, and had no previous experience or exposure to musical training. Following an evaluation by the music therapist, KH was recommended music therapy sessions for six months. The established goals of the music therapy were controlling the repetitive movements and behavior. During the therapy, no additional complementary or alternative medicine practices were administered, and no new medications were added or removed from his therapy plan.

### 2.2. Music Therapy Sessions

The music therapy was received from September 2018 to February 2019 at the Autism Academy of Jordan, which is one of the leading centers in Jordan, established in 2004 as the first center dealing particularly with autism, supervised and implemented by a music therapist who has a bachelor’s degree in music therapy. KH and the therapist met once a day for twelve weeks, followed by twice a week for the next twelve weeks. A session lasted forty-five minutes. The music therapy room was equipped with a piano, keyboard, and various percussion instruments.

During the six months of therapy, no other complementary, alternative, or conventional therapies were added or removed from his therapeutic plan.

#### Session Structure

A session had three main parts, as follows: warm-up, piano activities, and cool down.

- Warm-Up (10–15 min): dependent on the case

The warm-up part consisted of simple finger exercises on the piano to build fine motor skills and the incorporation of familiar melodies to encourage KH’s participation and engagement.

- Piano Activities (25 min)

This part involved an improvisation section followed by song learning. During improvisation, KH was encouraged to improvise on the piano, exploring different sounds and rhythms. This allowed him to express himself without the pressure of structured music. During song learning, the therapist introduced simple songs, focusing on repetition and pattern recognition as it is the way KH is keen to learn. After KH learnt to play short melodies, the complexity gradually increased as he gained some confidence.

- Cool Down (10 min)

KH played soft, calming music on the piano while reflecting on the session during this part. This helped him regulate his emotions and provided a peaceful end to the activity.

### 2.3. Progress Monitoring and Outcomes

**Figure 1:**
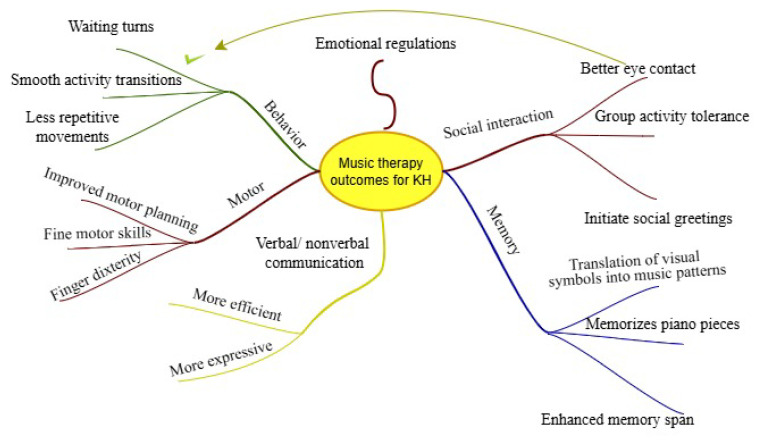
General psycho-social and cognitive outcomes of a child with ASD after six months of music therapy.

Noticeable improvements were experienced by the music and occupational therapists as well as his parents during and after the end of the planned therapy sessions. His progression on social interaction, verbal and nonverbal communication, emotional regulation, behavior, motor skills, and memory are summarized in Table 1 and Figure 1.

After the first three months of piano-focused music therapy, KH showed marked improvements in communication, social skills, emotional regulation, and behavioral adaptability. He became more vocal about his feelings and developed a deeper connection with the therapist through shared musical experiences. KH’s parents reported that he began exploring the piano at home, treating it as a close friend, where he could express his emotions without fear and often played pieces he had learned during therapy.

By the end of the six months of extensive therapy, KH exhibited musical acuity, showcasing the capacity to perceive pitch, rhythm, and tone. He is now able to engage in the facility’s scheduled activities; he plays the piano with assurance and performs significantly better than what is typically expected from an autistic child in a public setting. KH’s latent potential was significantly augmented during the music therapy sessions, profoundly influencing the quality of life for both KH and his parents. Furthermore, the sense of accomplishment derived from KH’s participation in the Autism Academy’s programs and events fosters pride in his parents and bolsters their confidence in his future opportunities.

**Figure 2:**
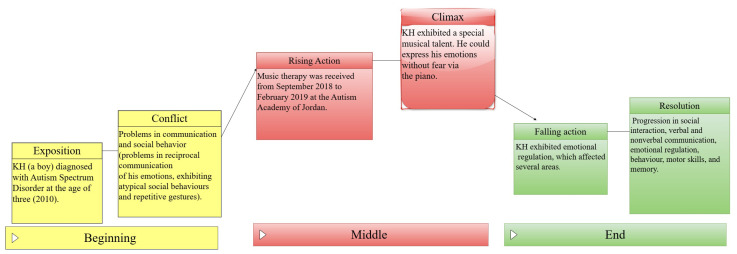
Summary of the music therapy’s progress for this case.

Figure 2 shows a summary of the music therapy’s progress for the presented case.

## 3. Discussion

Music therapy has been shown to greatly enhance social, emotional, and motivational development, particularly in individuals with autism who reportedly have a deficiency in the pleasant exchange of emotions, affect, and expressive feelings, which is regarded as a core characteristic of this population [13]. In contrast to other therapeutic interventions, music therapy serves as an interactive tool that enhances client engagement through its distinctive ability to relax the participant and to help them express their emotions and was purposefully designed to stimulate emotional involvement, responsiveness, and interpersonal initiative, potentially revealing musical talent [14].

Savant talents may be innate, manifesting from birth, or acquired, emerging after brain impairment or neuro-developmental disorders. The male-to-female ratio of savant syndrome is approximately 4:1; however, some researchers have reported ratios as high as 6:1 [15].

Our findings have substantiated the importance of music therapy in augmenting social reciprocity and fostering the development of daily life skills by affecting motor functioning and behavioral issues. The child in the present study, KH, exhibited increased discipline in everyday activities, as mentioned by his parents, such as following instructions, following the intervention. Moreover, after KH became able to participate in social events as a talented pianist and hearing his music, we can acknowledge that music therapy improved different aspects of his life, in addition to the quality of life of his parents.

Music’s unique acoustics possesses the capacity to affect the cerebral cortex across many brain regions in individuals with neurological diseases. It is possible to use music to enhance social relationships since the entire mind responds to music. Individualized music therapy plans enable children with autism to express their feelings and emotions through songs, melodies, and musical instruments [16]. Recent neuroimaging meta-analyses on brain responses to music listening have shown that it is significantly beneficial for patients with neurological diseases [17].

Music-based interventions, as mentioned in different studies, are important for the progress of new musical and social skills in children with ASD, as well as for fostering motor planning and confidence, and other aspects, as shown in our case [18].

Additionally, the frequent rhythm and pattern recognition, and memory recall aid in the development of cognitive abilities, including focus, attention, and memory, which in the case of autism, affect progress in other therapies such as occupational and speech therapy [17].

In the case of KH, the configuration of the piano keys created an intriguing structure that attracted his attention, as he exhibited a proclivity for systematization, described by a propensity to analyze, design, forecast, and regulate systems that follow rules [19].

The “Enhanced Perceptual Functioning” model supposes that an over-functioning of the brain can trigger parts of the brain that are responsible for the prevalence of special abilities. Another theory, “empathizing—systemizing”, suggests that the over-concentration on details in children with autism might allow them to better understand an object of interest, which therefore results in savant syndrome [20]. In our case, KH showed a propensity for engaging in systems governed by rules, and the configuration of the piano keys and the essence of musical rhythm provided an engaging framework that attracted his attention. Similarly, many children with ASD demonstrate a marked affinity for systemization. Consequently, these children may exhibit enthusiasm for interpreting piano patterns and imitating movements related to piano playing.

An interesting case similar to ours was described by Miller in 1989, in which a 5-year-old boy displayed remarkable proficiency as a young musician, which was beyond the expected abilities [21]. Moreover, Adam Ockelford presented several cases to elucidate the relationship between savant abilities and autism. He contended that many children with ASD experience sensory processing difficulties, which align with sensory integration theory. In instances where these children struggle to process visual stimuli effectively, they often compensate by engaging with auditory inputs, such as music. This occurs as a result of an ‘exceptional early cognitive environment’ (EECE), wherein children can interpret ordinary sounds, such as those produced by a clock, as musical rhythms. Additionally, echolalia, which is prevalent among children with ASD and involves the production of non-meaningful repetitive sounds, may collectively contribute to the development of exceptional musical abilities [22].

Finally, we can conclude, similarly to other studies’ consensus, that autistic talent or savant syndrome can be a result of intensive practice, training, and repetition, and in the case of music therapy, an individualized therapeutic plan for children with ASD might have a significant impact on their lives [23].

## 4. Limitations

Our study has certain limitations, including the restricted timeframe of the study; therefore, we do not know how much the music therapy contributed to the development of the boy’s abilities and what are the long-term effects of music therapy. Furthermore, the lack of a control group, given that this is a single case, restricts the generalizability of the results. It is essential to recognize that each child with ASD is a distinct case, where the child’s skills, weaknesses, interests, needs, family concerns, collaboration, and other aspects significantly influence the efficacy of the therapy.

## 5. Conclusions

This case study showed that music therapy can have a profound impact on children with autism, not only enhancing their ability to master a musical instrument and discover their hidden skills but also fostering growth across multiple areas of their lives like emotional regulation, social interaction, and cognitive processing, which improve their quality of life and affect progress in their occupational and speech therapy plans.

However, further research is needed to better understand the development of savant syndrome, especially in early intervention programs, and proposals for incorporating scientific technology into assessments of the direct effect of music on the brain and other physiological responses of children with ASD have been suggested. In addition, longitudinal studies are needed to help delve more into the long-term effect of music therapy on the capabilities and behavior of these children.

## Figures and Tables

**Table 1:** Improvements experienced by the therapists and parents.

Area of Improvement	Music Therapist’s Perspective	Occupational Therapist’s Perspective	Parents’ Perspective
**Social Interaction**	Increased ability to maintain eye contact during music sessions; engaged with the therapist in reciprocal music activities; by week 6, started engaging more with the therapist, sharing his thoughts about what to start with or what to play next.	Improved tolerance for being around other children during group gross motor activities, with reduced stress or anxiety in social settings while playing piano at public events.	Now initiates greetings and small interactions at family gatherings more frequently, with less stressful reactions.
**Emotional Regulation**	Increasing capacity to correctly link minor melodies with feelings of sorrow and major melodies with sentiments of joy; shows calm responses to slow-paced music, reducing instances of agitation during therapy; appears more relaxed post-session.	Remains calm longer when faced with tasks that previously triggered frustration, in addition to exhibiting less frequent impulsive reactions.	Seems less prone to becoming stressed or agitated at home and can follow a bedtime routine with fewer emotional seething by listening to soft piano music.
**Nonverbal Communication**	More frequent use of gestures (e.g., pointing to instruments or nodding) to express choices, indicating a desire to communicate without assistance.	Improved understanding of nonverbal cues, e.g., when the therapist pretends to be distracted or crying, by tapping on the table or on the therapist’s lap; increased use of hand signals as simple gestures to ask for something, like having more treats.	More expressive gestures and subtle actions (like little jumps) as indicators of happiness.
**Verbal Communication**	Started to use simple expressions related to music; initiated verbal exchanges during sessions; participates in vocal exercises; and attempts to occasionally use short phrases, like “more”, or the first letter of words.	Began using short sounds or phrases during activities that involve both speech and movement, like putting colored balls in the same color baskets with minimal assistance.	Tries to use words to request favorite activities, like saying “play” when he sees the piano at home.
**Behavioral Adaptability**	Shows increased patience by waiting his turn in mutual music activities, with music cues helping signal when to start and stop.	Greater ability to transition between tasks with minimal agitation; less irritation during hard tasks; follows simple routines when prepared in advance; ability to participate in public events and to play piano as part of a ceremony with confidence.	A smoother morning routine at home, with fewer difficulties transitioning from one activity to another; better sleep routine after combining piano time as part of a bedtime ritual.
**Motor Skills**	Engages with various musical instruments, showing precise movement between piano keys with normal pressure needed; finger dexterity and coordination on the piano enhanced, enabling him to play simple melodies with increased ease by week 10.	More able to accomplish tasks such as buttoning, brushing teeth, and tying his shoes, with less repetitive finger movement combined with tolerance of minor discomforts during complex motor tasks.	Less repetitive movements, which affect his eating manners, e.g., the spoon stays in his hand and no food is dropped, even with less verbal cues.
**Memory**	Ability to memorize piano pieces and remember musical patterns, rhythms, and melodies, and visual symbols and patterns.	In card-memorizing activities, became able to memorize up to 6 matching cards out of 10 without verbal cues from the therapist.	No memory difference mentioned.

## Data Availability

The original contributions presented in this study are included in the article.
